# Non‐enzymatic reaction of carnosine and glyceraldehyde‐3‐phosphate accompanies metabolic changes of the pentose phosphate pathway

**DOI:** 10.1111/cpr.12702

**Published:** 2019-10-19

**Authors:** Henry Oppermann, Claudia Birkemeyer, Jürgen Meixensberger, Frank Gaunitz

**Affiliations:** ^1^ Klinik und Poliklinik für Neurochirurgie Universitätsklinikum Leipzig AöR Leipzig Germany; ^2^ Institut für Analytische Chemie Universität Leipzig Leipzig Germany

**Keywords:** carnosine, glioblastoma, metabolomics, pentose phosphate pathway

## Abstract

**Objectives:**

Carnosine (β‐alanyl‐l‐histidine) is a naturally occurring dipeptide that selectively inhibits cancer cell growth, possibly by influencing glucose metabolism. As its precise mode of action and its primary targets are unknown, we analysed carnosine's effect on metabolites and pathways in glioblastoma cells.

**Materials and methods:**

Glioblastoma cells, U87, T98G and LN229, were treated with carnosine, and metabolites were analysed by gas chromatography coupled with mass spectrometry. Furthermore, mitochondrial ATP production was determined by extracellular flux analysis and reaction products of carnosine were investigated using mass spectrometry.

**Results:**

Carnosine decreased the intracellular abundance of several metabolites indicating a reduced activity of the pentose phosphate pathway, the malate‐aspartate shuttle and the glycerol phosphate shuttle. Mitochondrial respiration was reduced in U87 and T98G but not in LN229 cells, independent of whether glucose or pyruvate was used as substrate. Finally, we demonstrate non‐enzymatic reaction of carnosine with dihydroxyacetone phosphate and glyceraldehyde‐3‐phosphate. However, glycolytic flux from glucose to l‐lactate appeared not to be affected by the reaction of carnosine with the metabolites.

**Conclusions:**

Carnosine reacts non‐enzymatically with glycolytic intermediates reducing the activity of the pentose phosphate pathway which is required for cell proliferation. Although the activity of the malate‐aspartate and the glycerol phosphate shuttle appear to be affected, reduced mitochondrial ATP production under the influence of the dipeptide is cell‐specific and appears to be independent of the effect on the shuttles.

## 1 INTRODUCTION

In 1900, Gulewitsch and Amiradžibi characterized the first naturally occurring dipeptide carnosine (β‐alanyl‐l‐histidine), which was isolated from Liebig's meat extract.^1^ In human skeletal muscle, carnosine reaches a median concentration of about 20.0 ± 4.7 mmol per kg dry weight.^2^ Albeit human brain tissue contains more homocarnosine (0.16 mmol per kg dry weight^3^) than carnosine, the latter is found with higher concentrations (2.2 mmol per kg dry weight^4^) in the olfactory bulb. In the human body, carnosine levels are controlled by three genes. Human serum carnosinase (CNDP1) and cytosolic non‐specific dipeptidase (CNDP2) degrade the dipeptide, whereas carnosine synthase 1 (CARNS1), which is highly expressed in skeletal muscle, catalyses the formation of carnosine from β‐alanine and l‐histidine.^5^ Surprisingly, although the dipeptide is known since more than 100 years, its physiological role has not completely been resolved. Among the physiological functions described are pH‐buffering, chelation of heavy metal ions, scavenging of reactive oxygen species, protection from advanced glycation end‐products and protection from lipid peroxidation‐related cell damage (for a comprehensive review see Boldyrev et al^6^). In 1986, Nagai and Suda demonstrated that carnosine aside from “normal” physiological functions inhibits tumour growth in a mouse in vivo model.^7^ We and other groups confirmed this anti‐neoplastic effect using different types of cancer models, including glioblastoma, in vivo^8^, ^9^ and in vitro.^10^, ^11^, ^12^, ^13^ Since the anti‐neoplastic effect has been studied in more detail, a number of possibly involved signalling pathways have been suggested. Some reports point towards an inhibition of Akt phosphorylation 12, 14 and HIF‐1α signalling,^10^ and G1 and G2/M cell cycle arrest has been detected.^15^, ^16^ Furthermore, we and others reported that carnosine inhibits glucose‐dependent ATP production in glioblastoma cells.^11^, ^17^ However, the mechanisms responsible for carnosine's anti‐neoplastic effect are far from being understood. Additionally, a primary target has not been identified which especially pertains to carnosine's effect on glucose metabolism. Here, we analysed the metabolic profiles of tumour cells treated with or without carnosine in order to reveal whether primary targets of the dipeptide can be identified.

We decided to use glioblastoma cells in our study as this is the most frequent malignant tumour of the human brain^18^ with an incident of 3.21 new cases per 100 000 inhabitants in the United States. Under standard therapy consisting of maximal safe resection of the tumour, radiotherapy and adjuvant chemotherapy with temozolomide, median survival of patients is only 12‐15 months.^19^ Thus, there is an urgent need for additional therapeutic options to treat this highly malignant tumour of the central nervous system. Understanding the mechanisms by which carnosine exerts its anti‐neoplastic effect could well pave the way for the development of new therapeutic options.

## 2 MATERIALS AND METHODS

### 2.1 Chemicals and reagents

If not stated otherwise, all chemicals were purchased from Sigma‐Aldrich. Carnosine was kindly provided by Flamma S.p.A.

### 2.2 Cell culture

U87, T98G and LN229 cells were originally obtained from the ATCC and cultured in T‐75 culture flasks (Sarstedt AG & Co.) in DMEM/25 mM glucose, without pyruvate (Thermo Fisher Scientific), supplemented with 10% foetal bovine serum (FBS superior, Biochrom), 2 mM l‐alanyl‐l‐glutamine and antibiotics (Thermo Fisher Scientific) (further designated as “culture medium”) at 37°C and 5% CO_2_ in humidified air in an incubator. In order to confirm identity over long culture periods, cells were genotyped by STR analysis at the Genolytic GmbH using a PowerPlex^®^ 21 System (Promega) and cells were confirmed as the U87MG cell line from the ATCC.^20^

### 2.3 Growth rate determination

About 150 000 cells were seeded in culture medium (5 mL) in nine T‐25 culture flasks (Sarstedt). After 24 hours of incubation, cells in three flasks were detached using StemPro Accutase (Thermo Fisher Scientific) and cells were counted. Then, remaining cells were washed once with Hank's balanced salt solution (HBSS; 2 mL; 137 mM NaCl, 5.4 mM HCl, 0.41 mM MgSO_4_, 0.49 mM MgCl_2_, 0.126 mM CaCl_2_, 0.33 mM Na_2_HPO_4_, 0.44 mM KH_2_PO_4_, 2 mM HEPES, pH 7.4), followed by an incubation time of 72 hours in DMEM (1 mL) containing 25 mM glucose, 2 mM l‐alanyl‐l‐glutamine, N2 supplement (further designated DMEM N2) and supplemented with or without 50 mM carnosine. Then, cells were detached and counted, and the doubling time was calculated.

### 2.4 Determination of l‐lactate production rate

About 10^6^ cells were seeded in culture medium (2 mL) per well into 6‐well plates. After 24 hours of incubation, cells were washed once with HBSS (1 mL) and then further incubated with DMEM N2 and supplemented with or without 50 mM carnosine. After 0, 3, 6, 12, 24 and 32 hours, medium (5 µL) was collected and stored at −80°C until further use. l‐lactate was determined as described before.^21^ Absolute amounts of l‐lactate were calculated using a standard curve, and l‐lactate production rate was determined by calculating the l‐lactate production between three and six hours of incubation. In order to validate the significance of the data obtained, repeated measures one‐way ANOVA was performed using all time points.

### 2.5 Determination of glucose uptake rate

A total of 10 000 cells were seeded in culture medium (200 µL) in 96‐well plates (µClear, Greiner Bio‐One). After 24 hours of incubation, cells were washed once with HBSS (200 µL) and then further incubated with DMEM N2 and supplemented with or without 50 mM carnosine for 6 hours. Then, DMEM (30 µL) containing Hoechst 33 342 (10 ng/mL; Thermo Fisher Scientific) was added to each well and cells were counted using a Celigo Imaging Cytometer (Nexcelom Bioscience LLC) after an incubation for 30 minutes at 37°C. Afterwards, cells were washed once with HBSS (100 µL) and further incubated in the presence of 1 mM 2‐desoxy‐d‐glucose for 10 minutes. Formed 2‐desoxy‐d‐glucose‐6‐phosphate was determined using the glucose uptake Glo assay (Promega) according to the manufactures recommendations.

### 2.6 Metabolic profiling via GC‐MS

Metabolic profiling was performed as described previously.^21^ Briefly, 10^6^ cells were seeded in culture medium (2 mL) per well into 6‐well plates. After 24 hours of incubation, cells were washed once with HBSS (1 mL) and then further incubated with DMEM N2 (1 mL), supplemented with 0, 12.5, 25 or 50 mM carnosine. After incubation (see individual experiments for details), cell supernatants (10 µL) were collected from each well and immediately frozen at –80°C until further use. For the determination of intracellular metabolites, cells were placed on ice and briefly washed with ice‐cold HBSS. Immediately after washing, ice‐cold methanol (1 mL) was added to each well and metabolites were extracted for 24 hours on an orbital shaker at 8°C. Then, extracts were collected and wells were rinsed once with methanol. Afterwards, samples were evaporated to dryness using a speed vac (Maxi‐Dry Lyo, Heto‐Holten) and stored at −80°C until further use. Derivatization, gas chromatography coupled with mass spectrometry (GC‐MS) analysis and data evaluation were performed as described before.^21^, ^22^ If not stated otherwise, the abundance of a metabolite is defined by the peak area determined from the selected ion chromatogram of an experiment normalized to total cellular protein (µg).

### 2.7 Quantification of glyceraldehyde‐3‐phosphate and dihydroxyacetone phosphate

In order to investigate a possible reaction of carnosine with glyceraldehyde‐3‐phosphate (GA3P) and dihydroxyacetone phosphate (DHAP), GA3P (625 µM) or DHAP (625 µM) was incubated at 37°C for 4 hours in the presence of 0, 0.625, 1.25, 6.25 or 62.5 mM carnosine in triethanolamine buffer (50 mM triethanolamine; 5 mM MgCl_2_; pH 7.6; total volume: 75 µL). Then, the amounts of GA3P or DHAP were quantified according to Bergmeyer.^23^ Briefly, 1.5 µL of NADH solution (20 mM) was added to each reaction and the initial extinction at 340 nm was determined. Afterwards, enzyme mix (3.5 µL) was added to detect GA3P (1 U/µL triosephosphate isomerase; 0.085 U/µL glycerol‐3‐phosphate dehydrogenase diluted in triethanolamine buffer) or DHAP (0.085 U/µL glycerol‐3‐phosphate dehydrogenase, diluted in triethanolamine buffer). After an incubation of 30 minutes at 37°C, extinction at 340 nm was determined and the absolute amounts of GA3P and DHAP were calculated using a standard curve.

### 2.8 MS‐based analysis of carnosine reaction products

In order to identify a possible reaction product of carnosine and GA3P, GA3P (7.5 mM) was incubated in the presence or absence of carnosine (7.5 mM) in NH_4_^+^/CH_3_COO^−^ buffer (5 mM, pH 7.6) for 4 hours at 37°C. Then, the reaction mixture was diluted 1:12.5 in 30% acetonitrile/H_2_O and analysed by flow injection analysis on an ESI‐TOF micrOTOF (Bruker Daltonik) in positive mode (negative mode for GA3P detection) with an Agilent 1100 AS autosampler and otofControl 3.4 and HyStar 3.2‐LC/MS. Injection was performed with the following parameters: nebulizer (26.1 psi), dry gas flow (6 L/min), dry gas temperature (220°C), injection volume (5 µL), flow rate (0.1 mL/min) and eluent (methanol containing 0.1% formic acid). Mass selective detector was used with the following settings: mass range *m/z* 50‐1500, capillary exit 100 V, skimmer 1 50 V, hexapole 1 23 V, hexapole RF 70 Vpp, skimmer 2 23 V, lens 1 transfer 70 µs and lens 1 pre‐pulse storage 8 µs.

### 2.9 Determination of oxygen consumption rate (OCR) and extracellular acidification rates (ECAR)

For the determination of the OCR and the ECAR, 10 000 cells were seeded in 80 µL of culture medium in an XF96 cell culture microplate (Seahorse Bioscience). After 19 hours, cells were washed once with HBSS (1 mL) and then further incubated with DMEM (80 µL) containing 25 mM glucose, 2 mM l‐alanyl‐l‐glutamine, N2 supplement and 0 or 50 mM carnosine. After additional 6 hours of incubation, cells were washed twice with HBSS, followed by 1 h incubation at 37°C in 180 µL of Seahorse XF DMEM containing 25 mM glucose and 2 mM l‐alanyl‐l‐glutamine and 0 or 50 mM carnosine, or 180 µL HBSS containing 25 mM glucose or 5 mM pyruvate and supplemented with 0 or 50 mM carnosine. Then, Seahorse XF Cell Mito Stress Test was performed using an XF96 Extracellular Flux Analyzer according to the manufacturer. Briefly, after baseline determination, OCR was determined after the sequential addition of oligomycin A, FCCP and rotenone/antimycin A (each 1 µM). After the final measurement, Seahorse XF DMEM (30 µL) containing Hoechst 33342 (10 ng/mL) was added to each well and cells were counted using a Celigo Imaging Cytometer after an incubation of 30 minutes at 37°C. Data were analysed using the Seahorse Wave Desktop Software; OCR is reported as unit nanomole per minute and 10^6^ cells, and ECAR is reported as unit mpH per minute and 10^3^ cells.

### 2.10 Statistical analysis

Statistical analysis was carried out using SPSS (IBM; Version: 24.0.0.2 64‐bit). False discovery rate (FDR) was calculated according to Benjamini and Hochberg.^24^ For pairwise comparisons, a t test was performed. For multiple comparisons, a one‐way ANOVA with the Games‐Howell post hoc test was used. Principal component analysis (data were normalized to untreated control and auto‐scaled) and metabolite pathway analysis (settings: Over Representation Analysis: Hypergeometric Test; Pathway Topology Analysis: Relative‐betweenness Centrality; Pathway Database: Homo sapiens The Small Molecule Pathway Database) were performed using MetaboAnalyst.^25^ If not stated otherwise, experiments were carried out in 6‐tuplicate (independent experiments) and data are presented as mean ± standard deviation. Non‐overlapping confidence intervals (CI 95%), *P*‐value < .05 or FDR < 0.05 were presumed to be statistically significant.

## 3 RESULTS

### 3.1 Carnosine affects metabolic pathways connected to glycolysis

**Figure 1:**
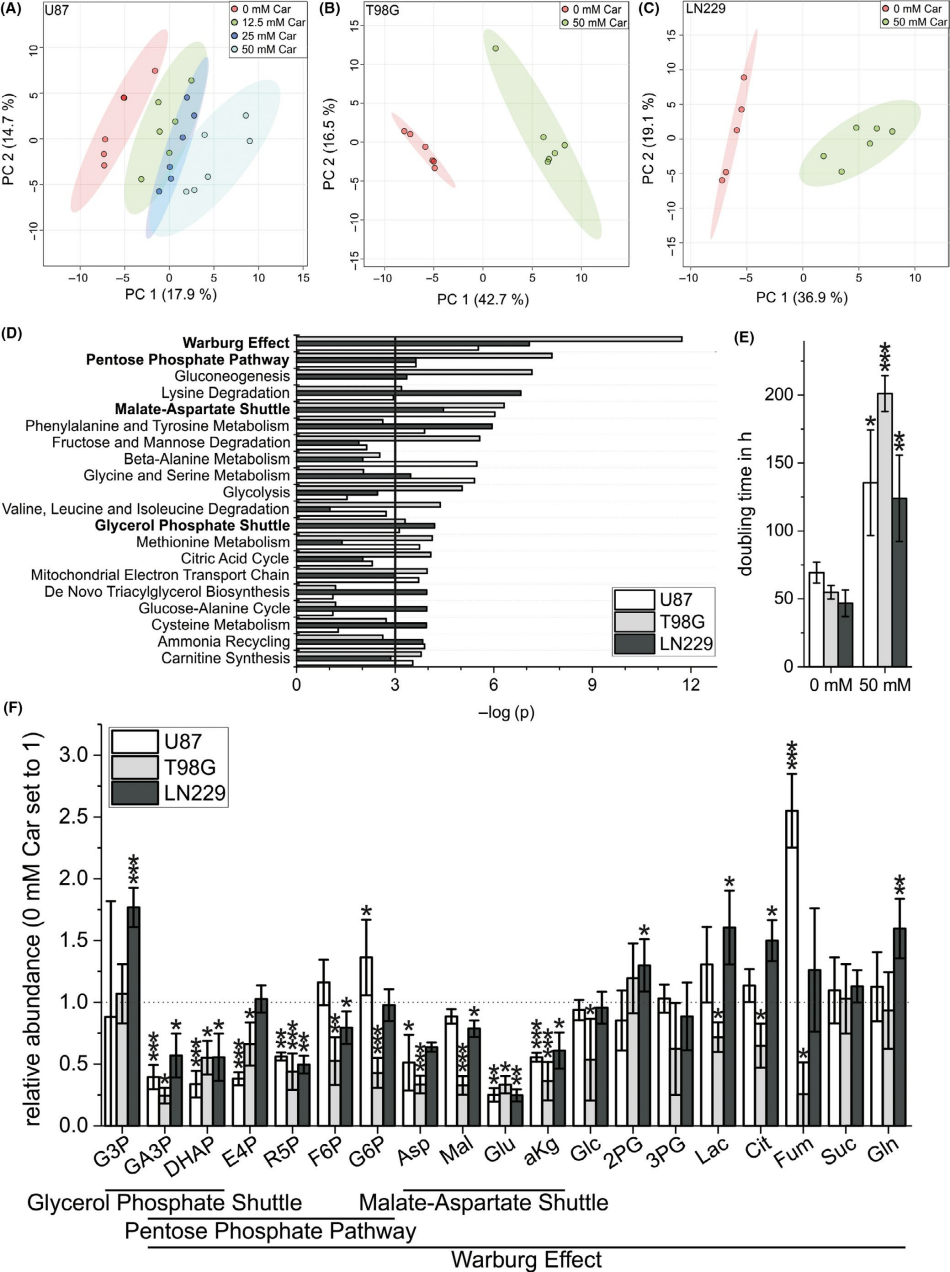
Impact of carnosine on glioblastoma cell metabolism. Principal component analysis of the metabolic profiles obtained from (A) U87 cells treated for 6 h with 0 mM (red), 12.5 mM (green), 25 mM (blue) or 50 mM (cyan) carnosine (Car) and (B) T98G and (C) LN229 cells treated for 6 h with 0 mM (red), 50 mM (green) Car. D, Pathway analysis using metabolites whose abundances were significantly (FDR < 0.05) changed by 50 mM Car. Bold line indicates a *P*‐value = .05. E, Doubling time of U87, T98G and LN229 cells treated with or without 50 mM Car (n = 3). F, Significantly, changed abundances of metabolites which belong to the metabolic pathways shown in (D). Data are presented as fold‐change compared to 0 mM Car (n = 6). 2PG, 2‐phosphoglycerate; 3PG, 3‐phosphoglycerate; aKg, α‐ketoglutarate; Asp, aspartate; Cit, citrate; DHAP, dihydroxyacetone phosphate; E4P, erythrose‐4‐phosphate; F6P, fructose‐6‐phosphate; Fum, fumarate; G3P, glycerol‐3‐phosphate; G6P, glucose‐6‐phosphate; GA3P, glyceraldehyde‐3‐phosphate; Glc, glucose; Gln, glutamine; Glu, glutamate; Lac, lactate; Mal, malate; R5P, ribose‐5‐phosphate; Suc, succinate. Level of significance is indicated as: ***P < .0005; **P < .005; *P < .05 vs. 0 mM Car

Previous studies indicated that carnosine affects cancer cell metabolism by influencing the glycolytic pathway.^11^, ^26^ As neither its mode of action nor targets of carnosine were known, we employed a targeted method using GC‐MS to study metabolites and pathways connected to glycolysis. Therefore, U87 glioblastoma cells were treated with FBS‐free medium containing different concentrations of carnosine (0, 12.5, 25 and 50 mM), followed by metabolite extraction and GC‐MS analysis after 6 hours of incubation. First, we asked whether the dipeptide has a general effect on cancer cell metabolism. Performing a principal component analysis (Figure 1A), we observed a significantly different metabolic profile already at a concentration of 12.5 mM carnosine. Increasing its concentrations for treatment resulted in an even better separation compared to the control cells, but confidence intervals of scores between cells treated with different concentrations of carnosine overlap, indicating a similarity between metabolic profiles under carnosine treatment. In order to distinguish between cell‐specific and general effects of the dipeptide, we also analysed intra‐ and extracellular metabolites in the glioblastoma cell lines T98G and LN229 treated with 50 mM carnosine for 6 hours. Similar to U87 cells, treatment of T98G and LN229 cells resulted in a clear separation in principal component analysis (Figure 1B,C). Next, we investigated which metabolic pathways are affected by the dipeptide using MetaboAnalyst. Therefore, abundances of intra‐ and extracellular metabolites of cells treated with 0 and 50 mM carnosine were compared by using a t test (for each cell line individually) and metabolites whose abundances exhibited an FDR < 0.05 were subjected to a pathway analysis (Figure 1D).

The metabolite set enrichment analysis revealed effects on several pathways, such as glycine and serine metabolism and lysine degradation with different probability among the cell lines. More important, four pathways, namely pentose phosphate pathway (PPP), malate‐aspartate shuttle, glycerol phosphate shuttle and Warburg effect (summarizes glycolysis, PPP and citric acid cycle), were significantly (*P* < .05) enriched in all three cell lines after carnosine treatment. The relative intracellular abundances of the detected metabolites of the corresponding pathways are shown in Figure 1F (for the complete results of the metabolic profiling see Figure S1).

The influence of carnosine on the PPP is highly interesting, as this pathway plays a critical role for proliferation, providing 5‐phosphoribosyl diphosphate for DNA and RNA biosynthesis.^27^ In accordance, treatment with 50 mM carnosine significantly increased the doubling time of U87, T98G and LN229 glioblastoma cells (Figure 1E).

In addition, the influence of carnosine on the glycerol phosphate shuttle and the malate‐aspartate shuttle suggests an inhibitory effect of carnosine on cellular redox homeostasis, as the malate‐aspartate shuttle contributes ~20% to the total respiratory rate, and its inhibition leads to reduced production of glutamate from glucose.^28^

### 3.2 Carnosine inhibits mitochondrial respiration independent of the malate‐aspartate and the glycerol phosphate shuttle

**Figure 2:**
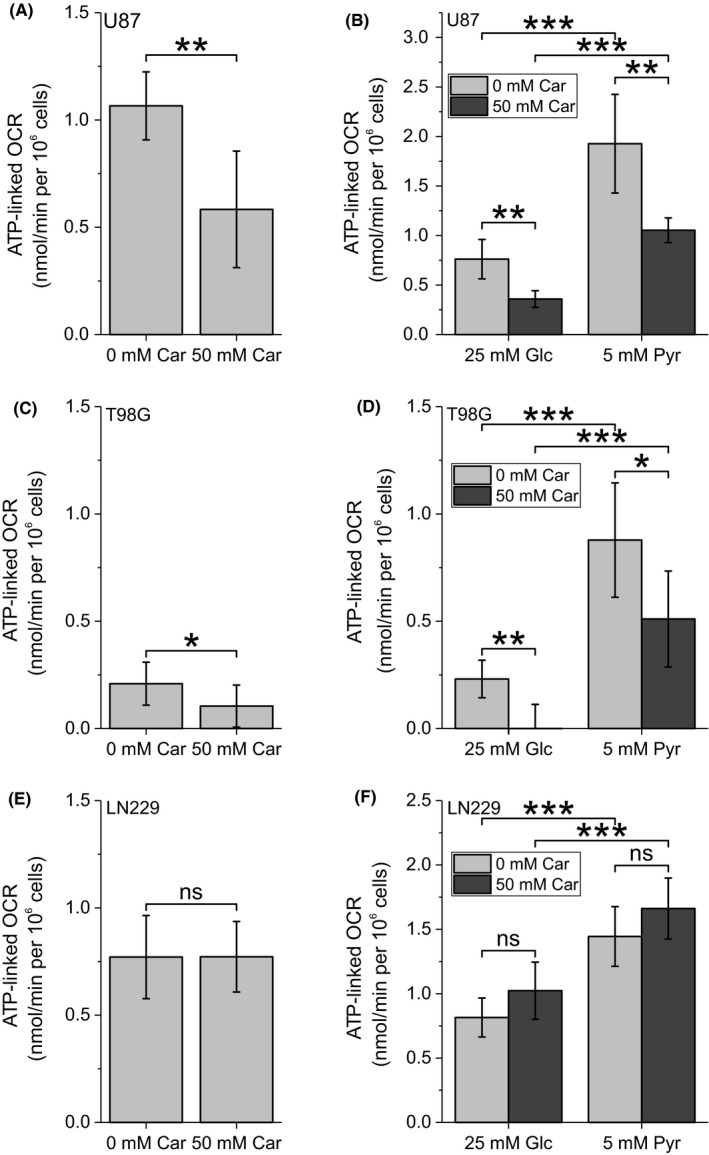
Carnosine inhibits mitochondrial ATP production in U87 and T98G cells. Determination of the ATP‐linked oxygen consumption rate (OCR) in (A) U87, (C) T98G or (E) LN229 cells treated with or without 50 mM carnosine (Car) for 6 h in the presence of DMEM with 25 mM glucose (Glc) and 2 mM l‐alanyl‐l‐glutamine (Ala‐Gln). Determination of the ATP‐linked oxygen consumption rate (OCR) in (B) U87, (D) T98G or (F) LN229 cells in HBSS containing 25 mM Glc or 5 mM pyruvate (Pyr) as the only energy source after 6 h treatment with 0 or 50 mM Car (n = 6‐10). Level of significance is indicated as: ***P < .0005; **P < .005; *P < .05; ns: *P* > .05

Our results suggest an inhibitory effect of carnosine on the glycerol phosphate shuttle and the malate‐aspartate shuttle which are required for mitochondrial ATP production. Hence, we investigated the influence of carnosine on mitochondrial respiration using a Seahorse XF analyser. In this experiment (Figure 2A,C,E; see also Figure S2), treatment with 50 mM carnosine significantly reduced the ATP‐linked oxygen consumption rate (OCR) of U87 and T98G cells but not that of LN229 cells (assayed in DMEM containing 25 mM glucose and 2 mM l‐alanyl‐l‐glutamine). Although reduced ATP‐linked OCR in U87 and T98G is in agreement with the assumption that it is caused by the dipeptide's influence on the aforementioned shuttles, the observation made in LN229 argues against this idea. Therefore, we determined the OCR in HBSS with pyruvate as the only substrate, as mitochondrial ATP production from pyruvate is independent of the malate‐aspartate shuttle and the glycerol phosphate shuttle.^29^ As seen in Figure 2B,D,F, ATP‐linked OCR was significantly higher in the presence of 5 mM pyruvate than in the presence of 25 mM glucose in all three cell lines. More important, we also observed reduced ATP‐linked OCR under the influence of pyruvate in U87 and T98G but not in LN229 (for the complete analysis of data obtained from the Seahorse experiments see Figure S3). Therefore, we conclude that carnosine's impact on mitochondrial respiration is cell‐specific and not necessarily dependent on its influence on the shuttle systems.

### 3.3 Glucose consumption and l‐lactate production

**Figure 3:**
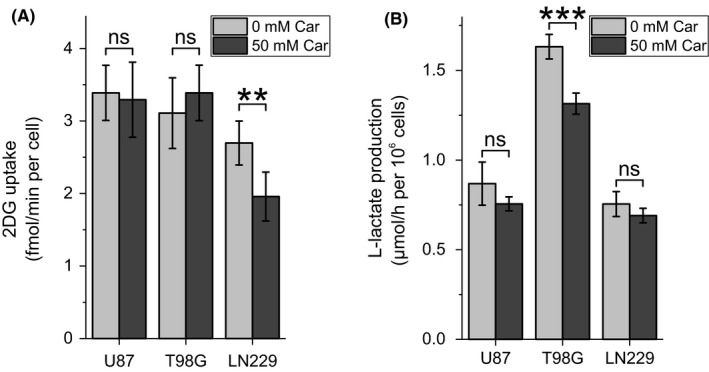
Influence of carnosine on glucose uptake and l‐lactate production. A, Cells were treated with or without 50 mM carnosine (Car) for 6 h. Then, 2‐desoxy‐d‐glucose (2DG) uptake was determined as a measure for the glucose uptake rate (n = 6). B, Cells were treated with or without 50 mM Car and extracellular l‐lactate was determined after 3 and 6 h to calculate l‐lactate production rate (n = 6). Level of significance is indicated as: ***P < .0005; **P < .005; ns: *P* > .05

In view of the observation that the abundances of DHAP and GA3P (Figure 1F) are reduced under the influence of the dipeptide, we wondered whether the flux from glucose to l‐lactate is also affected. Therefore, we determined glucose uptake rate (Figure 3A) and l‐lactate production rate (Figure 3B) under the influence of 50 mM carnosine. As can be seen in Figure 3A,B, uptake of 2‐desoxy‐d‐glucose was significantly reduced only in LN229 cells and l‐lactate production significantly reduced only in T98G cells (repeated measures one‐way ANOVA over all time points: *P* = .065, .0026 and .055 for U87, T98G and LN229, respectively).

### 3.4 Carnosine reduces the intracellular abundance of GA3P by a direct reaction with the metabolite

**Figure 4:**
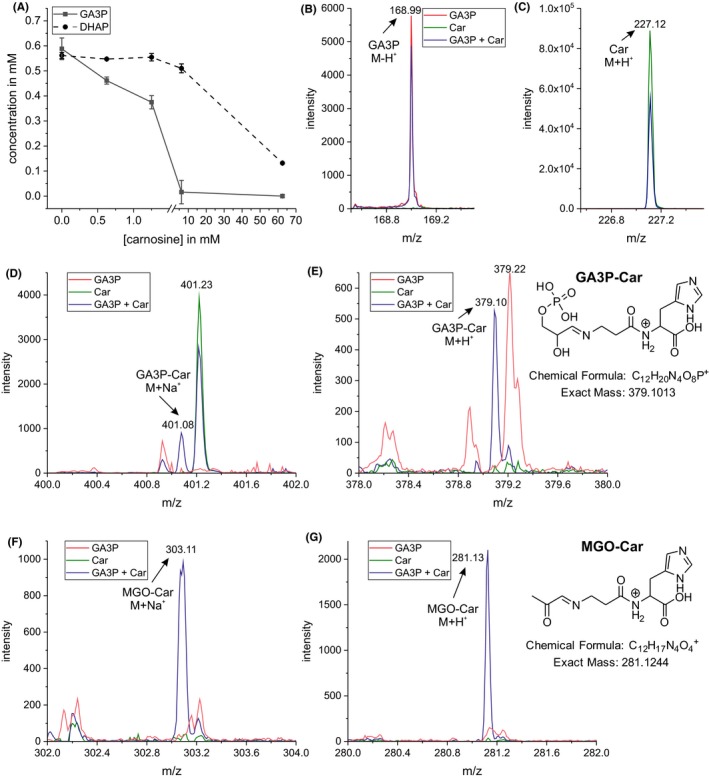
Carnosine reacts with glyceraldehyde‐3‐phosphate. A, Carnosine (Car) non‐enzymatically reacts with glyceraldehyde‐3‐phosphate (GA3P) and dihydroxyacetone phosphate (DHAP) (4 h of incubation; n = 3). Mass spectrometric analysis of reaction products of carnosine with GA3P and methylglyoxal (MGO). Intensity of (B) GA3P, (C) car, (D, E) GA3P‐Car, (F, G) MGO‐Car. Numbers indicate the corresponding *m/z*

Next, we investigated whether reaction of carnosine with DHAP and GA3P may be responsible for their decreased abundance in the presence of the dipeptide. Therefore, both triose phosphates were incubated in vitro in the presence of the dipeptide (Figure 4A). We observed that equimolar concentrations of carnosine resulted in a 1.3‐fold reduction of the GA3P concentration, and a ten‐fold higher concentration of the dipeptide resulted in a 36.5‐fold reduction of GA3P. We also observed a reduced concentration of DHAP in the presence of carnosine, but a 1.1‐fold reduction of DHAP required a ten‐fold molar excess of carnosine. This indicates that a non‐enzymatic reaction of carnosine with GA3P is responsible for the reduced abundance of the triose phosphates. To confirm a reaction of carnosine with GA3P, mass spectrometry was performed which detected two peaks with a mass of 379.1011 and 401.0829 (Figure 4D,E) referring to a reaction product of GA3P and carnosine ionized with H^+^ or Na^+^ (calculated mass: 379.1013 and 401.0833, respectively). Interestingly, we also detected the reaction product of methylglyoxal (MGO) and carnosine (Figure 4F,G), which was identified by the exact mass of 281.1264 (H^+^ ionization; calculated mass: 281.1244) and 303.1036 (Na^+^ ionization; calculated mass: 303.1064).

## 4 DISCUSSION

The naturally occurring dipeptide carnosine reduces tumour growth in vivo^8^, ^9^ and in vitro.^10^, ^11^, ^12^, ^13^ However, as pointed out in the introduction, neither the exact mechanisms behind this observation nor the primary targets of carnosine have been identified. Using mass spectrometry, we detected that carnosine non‐enzymatically reacts with GA3P in vitro similar to its previously described reaction with methylglyoxal.^30^ As we also see reduced intracellular abundances of GA3P and DHAP in cultured cells, we presume this reaction to be responsible for the reduced abundances of these triose phosphates. Surprisingly, the reduced abundance of these metabolites was not unequivocally accompanied by a decreased glucose uptake rate or by a decreased lactate release rate. Although one has to note that a precise determination of the glycolytic flux would require more powerful methods, such as ^13^C‐based metabolic flux analysis, this is an indication that the overall glycolytic flux is not decreased by the presence of the dipeptide. More importantly, the reduced abundances of GA3P and DHAP are accompanied by reduced abundances of metabolites associated with the PPP, the glycerol phosphate shuttle and the malate‐aspartate shuttle. The PPP is especially important for tumour cells as it provides nucleotides for nucleic acid synthesis and NADPH required for fatty acid synthesis and scavenging of reactive oxygen species (for review see^31^). Therefore, a decreased activity of this pathway will impair proliferation and defence against oxidative stress, and it has been discussed that the PPP could be in fact a potential target for cancer therapy.^32^

In previous investigations, the anti‐neoplastic of carnosine was shown to be associated with decreased ATP production. However, there have been different reports either pointing towards an effect on glycolysis, mitochondrial respiration or on both, depending on the cell type investigated.^11^, ^15^, ^17^, ^18^ As we observed a reduction of metabolites of the malate‐aspartate shuttle and the glycerol phosphate shuttle in all three cell lines, it is straightforward to assume that this observation is responsible for reduced mitochondrial ATP production as these pathways deliver cytosolic NADH to mitochondrial oxidation. However, using pyruvate as substrate, only cells from the lines U87 and T98G responded to the presence of the dipeptide with reduced ATP‐linked OCR but not cells from the line LN229. As LN229 cells do not respond with reduced ATP production even in the presence of glucose (Figure S3), aside from a predicted impairment of the shuttles, we assume that the shuttles may also be of less importance for ATP production in U87 and T98G cells. In addition, the cell‐specific response of mitochondrial ATP production to the presence of carnosine is highly interesting, pointing towards the possibility of a tumour‐specific reactivity. A cell‐specific response was also reported by Bao et al^17^ who demonstrated that carnosine inhibits the enzymatic activity of electron transport chain complexes in HeLa cells but not in SiHa cells. Therefore, future work should carefully evaluate in which cells carnosine affects ATP‐linked OCR to reveal the mechanisms behind this phenomenon. Nonetheless, carnosine impairs proliferation in all three lines examined. This in turn indicates that decreased mitochondrial ATP production is not as important for the anti‐neoplastic effect of the dipeptide as a decreased activity of the PPP.

Up to now, several suggestions have been made on how carnosine may influence tumour cell growth (for a review see^33^). A recent report analysing a potential role of the PI3K/Akt/mTOR signalling pathway in U87 and T98G cells demonstrated that the inhibitory effect of carnosine is independent of this pathway, although cells from the line U87 exhibited a decreased phosphorylation of Akt under the influence of the dipeptide.^14^ Whether the effect on Akt is caused by the influence of carnosine on metabolites or mitochondrial respiration is difficult to resolve as these interactions are highly complex (for review see^34^). At this point, it should also be emphasized that care has to be taken to compare the dipeptide's effect on different cancer cell types. Whereas in glioblastoma cells no effect on mTOR signalling was detected,^14^ Zhang et al reported an inhibitory effect on mTOR/p70S6K‐signalling in gastric carcinoma.^12^ As increased GA3P levels lead to activation of mTOR,^35^ a reduction of GA3P could be responsible for an inhibitory effect. To solve the riddle why glioblastoma cells behave differently with regard to mTOR signalling would require further investigations on how phosphorylation/dephosphorylation of mTOR is controlled in different cells. However, both observations taken together argue against the notion that mTOR is a direct target of carnosine, pointing towards an upstream target such as GA3P.

As it is reported that carnosine suppresses HIF‐1α signalling,^10^, ^36^ it is also tempting to speculate that inhibition of this pathway, which controls the expression of glycolytic enzymes, is responsible for the effects observed in this study. However, there are also reports that the dipeptide may increase HIF‐1α activity accompanied by reduced tumour growth and extracellular acidification.^37^ In order to resolve whether HIF‐1α signalling is involved in carnosine's anti‐neoplastic effect certainly requires further investigations. At this point, it should also be noted that the influence of metabolites on signalling is still far from being understood.^38^

Our observation that all three lines responded to the presence of carnosine with decreased activity of the PPP and with decreased proliferation when supplied with glucose is in agreement with more recent interpretations of the Warburg effect. Warburg's observation that cancer cells exhibit a high rate of glycolysis even in the presence of oxygen (aerobic glycolysis) has long been debated. Today, the most accepted interpretation trying to explain this phenomenon is that aerobic glycolysis, despite its low efficiency in ATP yield per molecule glucose, strongly supports macromolecular synthesis (for review see^39^). Therefore, the reduced abundances of metabolites such as GA3P, DHAP, E4P or R5P can be interpreted as a direct effect of carnosine on the Warburg effect. Noteworthy, our results also demonstrate that the cells from all three lines are able to produce ATP in the presence of pyruvate and in the absence of glucose confirming that mitochondria are not defective in their ability to carry out oxidative phosphorylation (OXPHOS). However, as carnosine interferes with OXPHOS in U87 and T98G cells but not in LN229 cells, mitochondrial physiology may be different in different cells. This can even be the case within one tumour. Janiszewska et al, for example, demonstrated that especially glioblastoma cancer stem cells strongly appear to be dependent on OXPHOS but not on glycolysis.^40^ As there is now emerging evidence that cancer cells can acquire a high metabolic plasticity by their hybrid glycolysis/OXPHOS phenotype,^41^ and it is interesting that carnosine is able to interfere with both pathways. Hence, our observations strongly demand further research into carnosine's effect on OXPHOS, as metabolic plasticity may be associated with metastasis and therapy‐resistance.

Considering using carnosine for the therapy of glioblastoma or other types of cancer, the question has to be answered whether intracellular concentrations of carnosine could be reached that are able to inhibit the PPP by reaction with GA3P. The experiments presented in Figure 4A demonstrate that a concentration of >1 mM carnosine should be reached to reduce the available amount of GA3P to <50%. This is at least in the range of carnosine concentrations determined in kidney tissue of CN1 transgenic mice which were supplemented with a bolus of 200 mg carnosine.^42^ In these experiments, carnosine concentrations reached ~0.5 mmol/kg 8 hours after supplementation. More interestingly, the authors could also demonstrate that in mice receiving a CN1 inhibitor, tissue concentrations increased ~7‐fold, which is an interesting aspect considering carnosine as a therapeutic. In this context, one should also be aware that in the experiments of Qiu et al,^42^ carnosine was supplemented only once and tissue concentrations were determined 8 hours later. However, in a clinical set‐up carnosine might be supplemented repeatedly. As demonstrated by the experiments of Blancquaert et al,^43^ daily administration of the carnosine moieties β‐alanine and l‐histidine over 23 days results in a continuous increase in muscle carnosine concentrations. This observation points towards the possibility that the dipeptide's tissue concentration might continuously be increased after repeated supplementation during cancer therapy.

In conclusion, our investigation demonstrates that carnosine's anti‐neoplastic effect is accompanied by reduced abundances of several metabolites associated with glycolysis and pathways dependent on it. We suggest that a non‐enzymatic reaction of GA3P with the dipeptide is responsible for its reduced abundance that in turn leads to a decreased activity of the pentose phosphate pathway required for proliferation. However, we also observed an inhibitory effect on mitochondrial ATP production which is cell‐specific and independent of carnosine's effect on glycolytic metabolites.

## CONFLICT OF INTEREST

The authors declare that they have no potential conflict of interest.

## AUTHOR CONTRIBUTIONS

HO performed the experiments and analysed the data. CB carried out the mass spectrometry measurements and identified the reaction products. JM revised the manuscript. HO and FG designed the study and wrote the manuscript. All authors reviewed and approved the manuscript.

## Data Availability

The datasets generated in this study are available from the corresponding author on reasonable request.
