# Supplementary figures and images for: Non‐enzymatic reaction of carnosine and glyceraldehyde‐3‐phosphate accompanies metabolic changes of the pentose phosphate pathway
Source: Cell Prolif. 2019 Oct 19;53(2):e12702. doi: 10.1111/cpr.12702 (PMC7046307; doi:10.1111/cpr.12702)

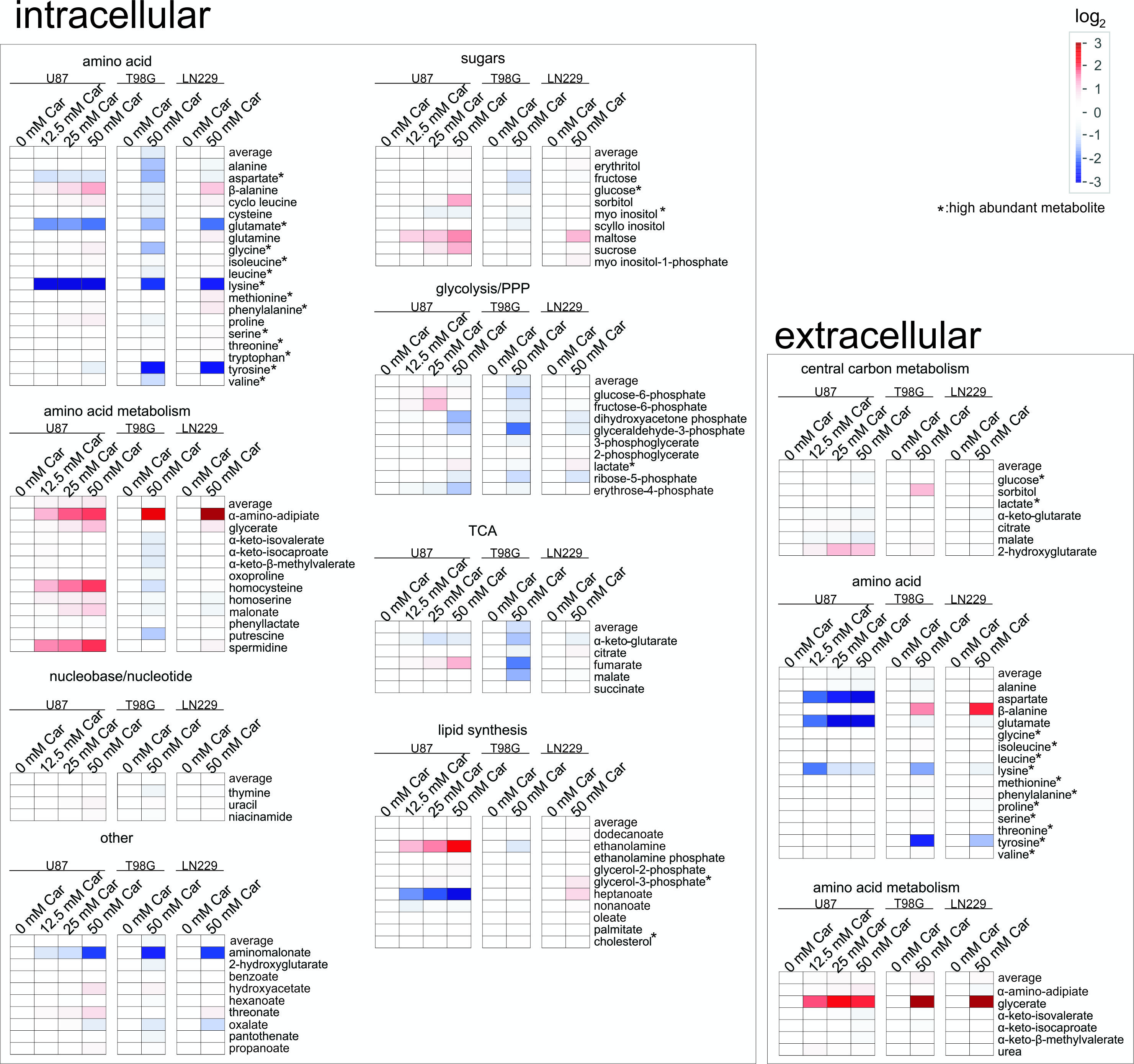

Supplement: Supplementary file 1 [file CPR-53-e12702-s001.jpg]

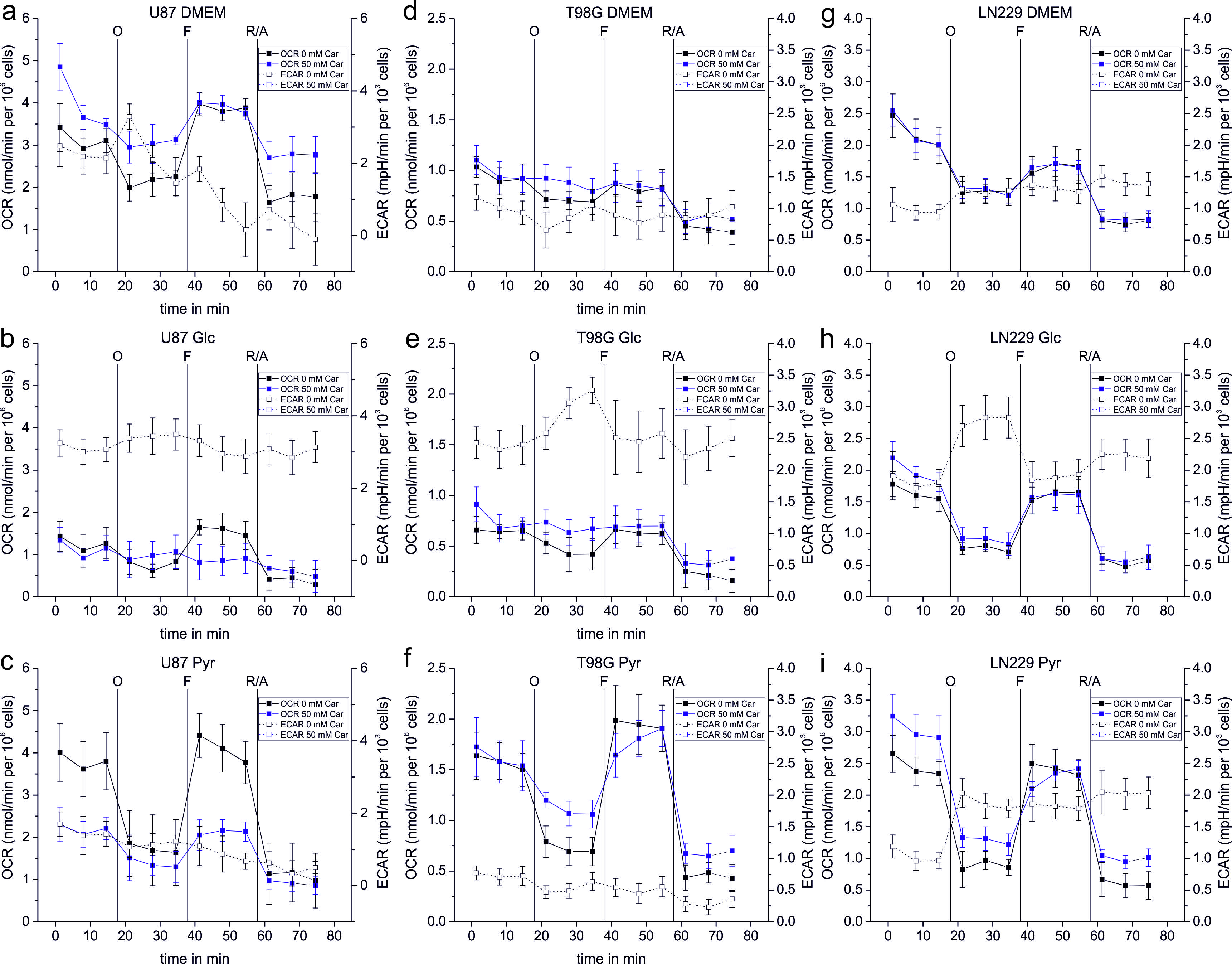

Supplement: Supplementary file 2 [file CPR-53-e12702-s002.jpg]

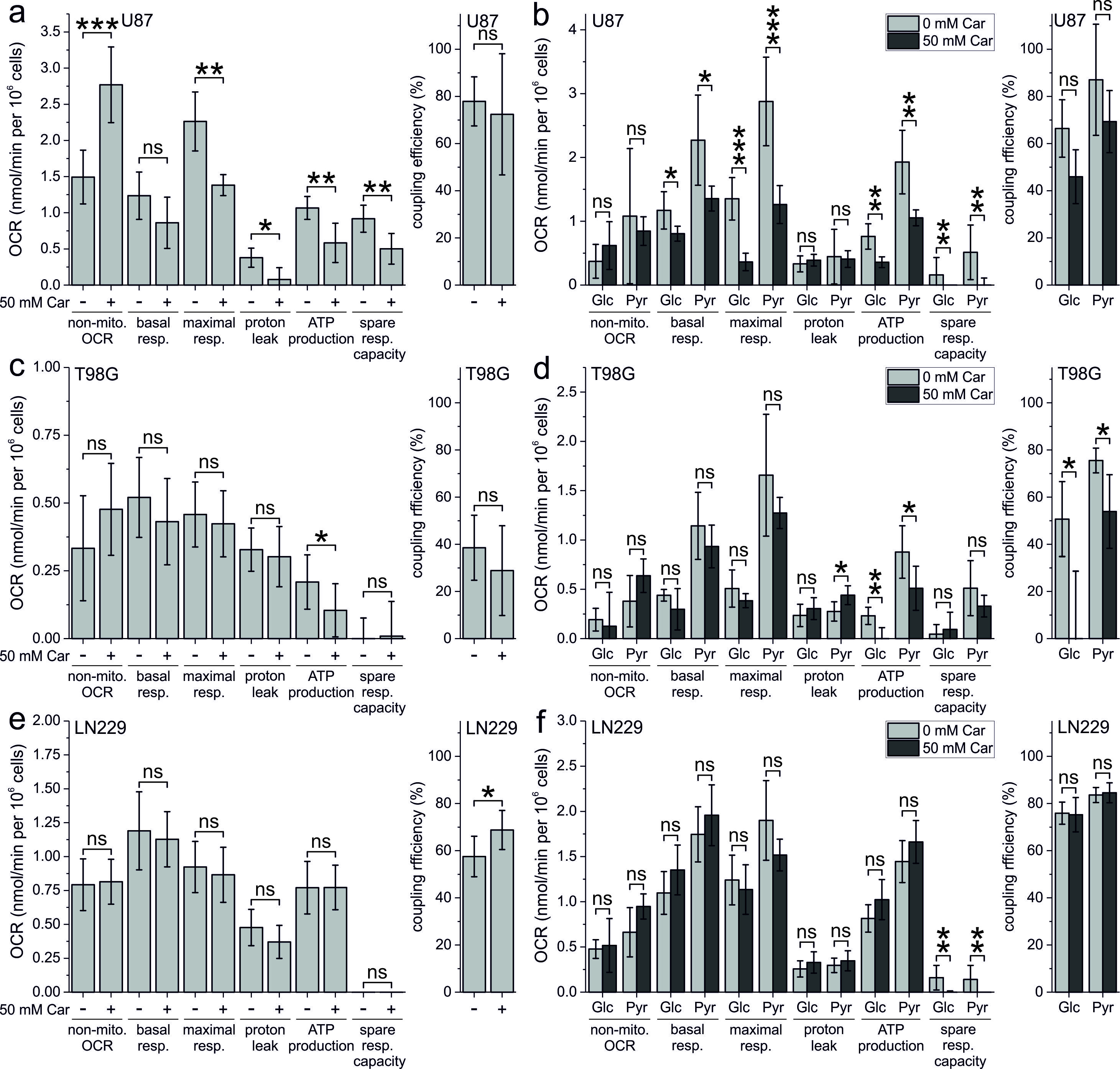

Supplement: Supplementary file 3 [file CPR-53-e12702-s003.jpg]
