# Supplementary material for: Non‐enzymatic reaction of carnosine and glyceraldehyde‐3‐phosphate accompanies metabolic changes of the pentose phosphate pathway
Source: Cell Prolif. 2019 Oct 19;53(2):e12702. doi: 10.1111/cpr.12702 (PMC7046307; doi:10.1111/cpr.12702)
Supplement: Supplementary file 4 [file CPR-53-e12702-s004.docx]

**Supplementary information**

**Supplementary Figure 1|** Comparative metabolic profiling of U87, T98G and LN229 cells cultivated in the presence of different concentrations of carnosine. A detailed overview of analyzed metabolites as shown in Figure 1 is presented as heat map. Fold changes (log_2_-transformed) of intra- and extracellular cellular metabolite abundances are indicated with regard to the carnosine (car) concentration employed (0 mM; set to log_2_(1)=0; n=6 independent samples per condition). Identified metabolites were grouped dependent on their metabolic role: TCA: metabolites associated with the tricarboxylic acid cycle; lipid synthesis: metabolites associated with lipid synthesis; glycolysis/PPP: metabolites of glycolysis and pentose phosphate pathway; nucleobase/nucleotides; sugars; amino acids; amino acid metabolism: metabolites associated with amino acid metabolism; other: other metabolites not unequivocally associated with the other pathways indicated. The average of the fold-changes from the metabolites in each group is indicated as “average”. Highly abundant metabolites are marked with * indicating a non-linear relation between metabolite concentration and abundance.

**Supplementary Figure 2|** Regulation of oxygen consumption rates (OCR) and extracellular acidification rates (ECAR) after subsequently injection of oligomycin A (O), FCCP (F) and rotenone/antimycin A (R/A). U87 (**a**, **b**, **c**), T98G (**d**, **e**, **f**) or LN229 (**g**, **h**, **i**) cells treated with or without 50 mM carnosine (Car) for 6 hours in the presence of DMEM with 25 mM glucose (Glc), N2 supplement and 2 mM L-alanyl-L-glutamine (Ala-Gln). OCR and ECAR were assayed in DMEM supplemented with 25 mM Glc and 2 mM Ala-Gln (**a**, **d**, **g**), HBSS containing 25 mM Glc (**b**, **e**, **h**) or 5 mM pyruvate (Pyr) (**c**, **f**, **i**) (n=6-10). Note that negative ECAR levels are not shown. This pertains especially for the determination ECAR in the presence of carnosine which exhibits pH-buffer properties.

**Supplementary Figure 3|** Analysis of the Seahorse XF experiment shown in supplementary Figure 2. U87 (**a**, **b**), T98G (**c**, **d**) or LN229 (**e**, **f**) cells treated with or without 50 mM carnosine (Car) for 6 hours in the presence of DMEM with 25 mM glucose (Glc), N2 supplement and 2 mM L-alanyl-L-glutamine. Oxygen consumption rates (OCR) were determined after subsequent injection of oligomycin A, FCCP and rotenone/antimycin A in DMEM supplemented with 25 mM Glc and 2 mM Ala-Gln (**a**, **c**, **e**), HBSS containing 25 mM Glc or 5 mM pyruvate (Pyr) (**b**, **d**, **f**) (n=6-10). Values for mitochondrial respiration were calculated using the Seahorse Wave Desktop Software. Abbreviations: mito.: mitochondrial; resp.: respiration
